# Non-canonical gene amplifications facilitate adaptive evolution in bacteria

**DOI:** 10.1038/s41564-026-02415-2

**Published:** 2026-07-06

**Authors:** Idan Yelin, Roy Kishony

**Affiliations:** 1 Faculty of Biology, Technion—Israel Institute of Technology, Haifa, Israel; 2 Faculty of Computer Science, Technion—Israel Institute of Technology, Haifa, Israel; 3 Faculty of Biomedical Engineering, Technion—Israel Institute of Technology, Haifa, Israel; 4 Center for Integrated Solutions for Infectious Diseases (CISID), Broad Institute of MIT and Harvard, Cambridge, MA USA

**Keywords:** Bacterial genetics, Transposition, Mobile elements, Experimental evolution

## Abstract

Gene amplification, a common route to bacterial adaptation, often occurs through recombination between two copies of an insertion sequence (IS) element flanking a genomic region. Alternative non-canonical structures have also been proposed, in which a duplication is formed by a single IS element whose two ends join two distant chromosomal loci. However, the prevalence of such non-canonical structures and their role in bacterial adaptive evolution remain unclear. Here we developed AmpliFinder, a computational tool that uses short-read sequencing data to systematically identify pairs of IS–chromosome junctions that correspond to the two ends of the same IS element yet map to distant genomic loci flanking amplified regions. Applying AmpliFinder to 10,347 laboratory-evolved *Escherichia coli* and *Acinetobacter baumannii* isolates, we identified 113 distinct de novo IS-associated amplifications and found that non-canonical amplifications are the most abundant mode of amplification. We validated the inferred architectures using ultra-long-read sequencing and propose a model for non-canonical amplification formation supported by the observation of nested intermediate structures. Quantifying enrichment for antibiotic-resistance genes in amplicons, we find that non-canonical amplifications more effectively and narrowly amplify genes under selection. These results highlight the role of non-canonical IS-based amplifications in the adaptive evolution of bacteria.

## Abstract (web summary)

Transposon-associated amplifications are dominated by a previously underappreciated architecture, where the transposon is found only between, not flanking, the amplicons. These amplifications facilitate the evolution of bacteria and their adaptation to antibiotics.

## Main

Gene amplification is a key mechanism in bacterial evolution. Generating an array of multiple copies of a given chromosomal region (amplicon), this process can transiently increase the expression of enclosed genes, allowing rapid, yet reversible, adaptation to environmental pressures^1–7^. In clinical isolates, gene amplifications have been implicated in drug resistance and heteroresistance^8–10^. In laboratory-evolved strains, gene amplifications have been shown to underlie adaptation to various environmental challenges, including metabolic^11–17^, ecological^18,19^ and antibiotic stressors^20–22^. Analogous to clinical isolates, when challenged by antibiotics in a laboratory setting, mutants carrying amplifications of regions containing antibiotic-resistance genes, or the drug targets, often emerge^9,23–32^.

**Fig. 1:**
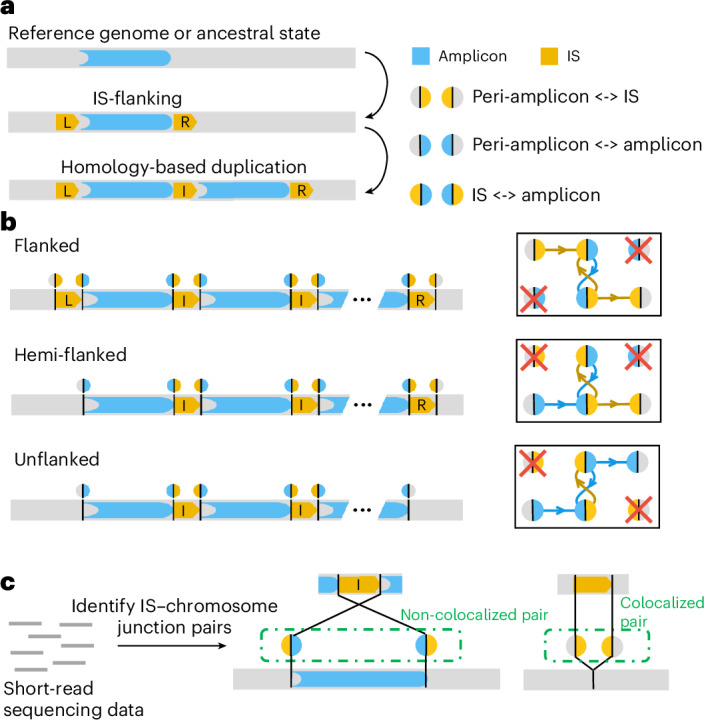
IS-associated amplification structures and their sequence junctions. **a**, A natively single-copy region (blue) in a chromosome (grey) can be amplified in association with IS elements (yellow). Canonically, once such a single-copy region is flanked by two ISs (either pre-existing in the genome or moving there via transposition events), it can duplicate and further replicate via homologous recombination, creating a tandem repeat of an amplicon separated by internal ISs (‘I’) and which is also necessarily flanked by ISs on both sides (‘L’, ‘R’ ISs; ‘flanked’ configuration). **b**, Non-canonical amplification cassettes, presumably created via replicative transpositions (Extended Data Fig. 1), are lacking either one or both of these flanking ISs, creating a ‘hemi-flanked’ or ‘unflanked’, structure. Right: These three IS-associated amplification structures can be described as paths (coloured arrows) between a unique subset of four out of six possible type-determining genomic junctions (circles; crossed out signifies junction absence). **c**, Identifying IS-associated amplicons based on non-colocalized IS–chromosome junctions. By identifying pairs of sequence junctions between the two sides of an IS and two distant positions in the bacterial genome (non-colocalized IS–chromosome junction pairs, Methods), as opposed to colocalized pairs characteristic of a simple transposition, we define the boundaries of potential IS-associated amplicons.

**Extended Data Fig. 1:**
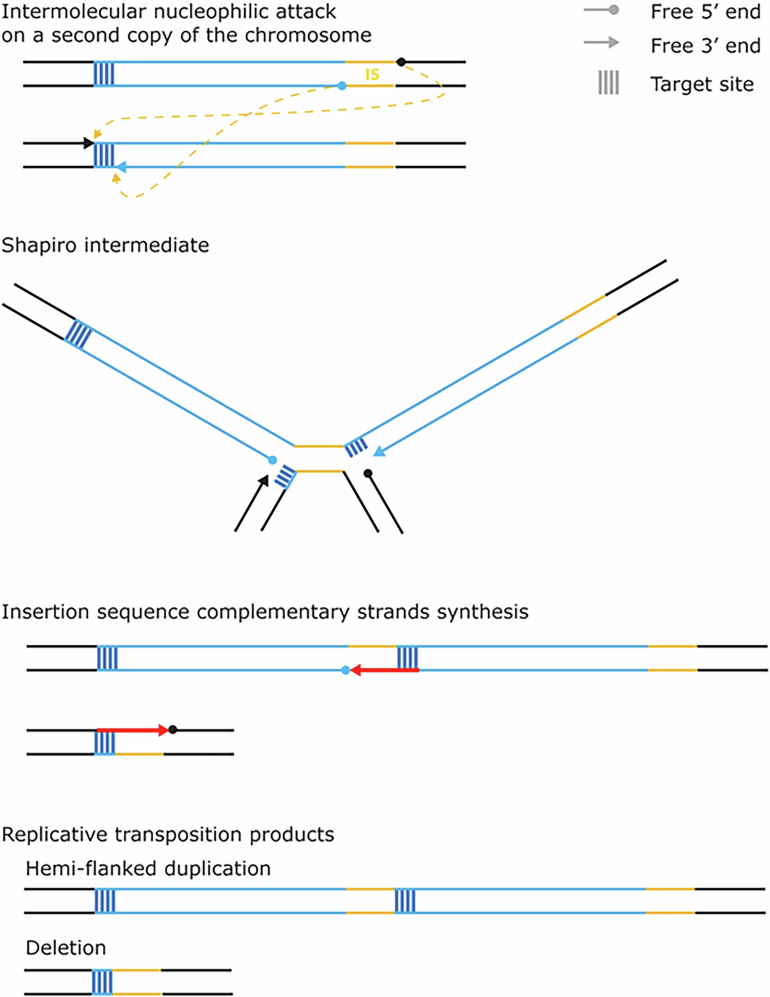
A suggested inter-chromosomal replicative-transposition based model for the formation of hemi-flanked amplifications. A replicative-transposition event in which the IS replicates into a second copy of the same chromosome may result in a hemi-flanked duplication (amplicon in light blue)^35^. The 3′ ends of the IS element are cleaved and attack a target site at the other side of the amplicon (blue). A Shapiro intermediate forms in which the IS element bridges between the two copies of the chromosome, being connected with its 5′ ends to the original site, and with its 3′ ends to the target site. The complementary strands of the IS element are synthesised starting at the free 3′ ends generated at the target site, resulting in two products, one with two copies of the amplicon and one with none.

Once initiated, amplification can further proceed through homologous recombination between copies of the amplified region, yet initiation of this process requires first a step of duplicating a natively single-copy region^4,33,34^. While this initial duplication can be created by a range of mechanisms^35–39^, a common mechanism is through insertion sequence (IS) elements, minimal transposable elements^40–43^. Such transposable elements, which are known drivers of bacterial evolution^31,40,44,45^, are often seen flanking the regions to be amplified, providing homology for duplication by homologous recombination^1,46–53^. These flanking IS elements either may pre-exist on both sides of the region to be amplified, or can move there through transposition events^1,46–53^. An amplification array initiated by homologous recombination between such bordering IS elements is expected to have a structure in which consecutive copies of the amplicon are separated by ‘internal’ IS elements and the entire array also has ‘flanking’ IS elements on its two sides (‘flanked’ architecture, Fig. 1a). Yet, in rare cases, amplification arrays in which amplicons are still separated by ISs but the array is bordered only by an IS element from one side (‘hemi-flanked’)^35,54–57^, or even not at all (‘unflanked’)^35,54,57–59^, have also been identified. While a replicative transposition-based mechanism may directly create hemi-flanked amplifications^35^ (Extended Data Fig. 1), how unflanked amplifications are formed and whether they require a single or multiple molecular steps, is less understood^54,59–61^. More generally, it remains unclear whether such non-canonical structures are a rare molecular nuance or a general facilitator of adaptive evolution.

Genomically identifying and distinguishing among the different types of IS-associated gene amplification architectures is challenging. While long-read sequencing can, in principle, capture the architecture of amplified regions and associated IS elements, ultra-long reads are needed to cover the entire length of an amplification array. Furthermore, long-read datasets are not commonly available for lab-evolved bacterial isolates. For the more commonly available short-read sequencing data, several computational methods have been developed to detect amplifications or other large-scale genomic rearrangements^62–64^ and to detect regular, but not replicative, IS transpositions. Amplifications are resolved by identifying high-coverage regions, possibly flanked by new sequence junctions^12,65,66^. Regular IS transposition events are resolved based on identifying pairs of colocalized IS–chromosome sequence junctions: the two sides of the IS form new sequence junctions with the chromosome, which point in opposite directions from the same chromosomal locus (‘same-locus IS’)^67,68^. Such approaches thereby cannot detect ISs created via replicative transpositions, or the internal IS elements separating consecutive copies of an amplicon; the IS–chromosome junctions corresponding to the two ends of such ISs are non-colocalized, pointing at opposite directions from two different loci of the ancestral chromosome (‘locus-joining IS’). Furthermore, systematic methods for linking transposition events with gene duplication have been lacking. Therefore, the genomic architecture of gene amplification and the role of IS elements in facilitating these evolutionarily important adaptive steps remain unclear.

Here, by systematically identifying colocalized and non-colocalized IS–chromosome junction pairs bordering amplified genomic regions in a large short-read sequencing dataset of laboratory-evolved bacteria, we reveal a range of de novo-occurring amplification events, finding that non-canonical amplifications are, in fact, the dominant, most abundant, mode of amplifications. We validate the inferred architectures, which cannot be unambiguously called by short reads, using ultra-long-read sequencing. Analysing intermediate nested amplification structures, we suggest a model for the formation of non-canonical amplifications by successive replicative transposition events. Contrasting amplification events of distinct architectures, we find that non-canonical amplifications are shorter, have higher copy numbers and are associated with specific types of IS elements. Quantifying enrichment for antibiotic-resistance genes in amplicons identified in bacteria adapted to antibiotic drugs, we find that non-canonical amplifications more effectively and narrowly amplify genes under selection. Lastly, we show experimentally how a non-canonical duplication facilitates evolvability driven by rapid amplification.

## Identifying IS-associated amplification architectures

The flanked, hemi-flanked and unflanked IS-associated amplification architectures can be distinguished by the presence and absence of colocalized and non-colocalized IS–chromosome sequence junction pairs (Fig. 1). For a given amplicon, potentially flanked by ISs, we consider the four possible IS–chromosome junctions: two connecting the right and left sides of the IS to the left and right sides of the amplicon (IS–amplicon, amplicon–IS) and two connecting each side of the IS to the chromosomal regions peripheral to the amplification array (IS–periamplicon, periamplicon–IS). The flanked amplification architecture is expected to have all four junctions whose colocalized and non-colocalized pairing defines the three expected ISs: the left- and right-flanking ISs are defined by the two colocalized pairs of IS-to-amplicon and IS-to-periamplicon junctions (periamplicon–IS ↔ IS–amplicon and amplicon–IS ↔ IS–periamplicon), and the internal locus-joining IS is defined by the non-colocalized pair of the two IS-to-amplicon junctions (amplicon–IS ↔ IS–amplicon; Fig. 1a,b; there is a fourth possibility of an IS connecting the two bordering chromosomal regions, periamplicon–IS ↔ IS–periamplicon, corresponding to the deletion of the amplicon, not shown). By contrast, hemi-flanked or unflanked amplifications, lacking one or both of the flanking ISs, are expected to be void of one or both of the IS–periamplicon junctions (Fig. 1a). In parallel, the distinction between these three structures is also reflected by the presence and absence of the ancestral amplicon–periampliconial chromosome junctions (periamplicon–amplicon and amplicon–periamplicon): while in the flanked architecture, these junctions are superseded by the flanking ISs, one or both of these ancestral junctions should remain intact in the hemi-flanked and the unflanked, respectively (Fig. 1a). Consequently, the three different architectures can be identified by the presence and absence of a ‘type-determining’ set of six junctions: two amplicon-to-periamplicon junctions (periamplicon–amplicon, amplicon–periamplicon), two IS-to-amplicon junctions (IS–amplicon, amplicon–IS) and two IS-to-periampliconial chromosome junctions (IS–periamplicon, periamplicon–IS; Fig. 1a).

To identify IS-flanked, hemi-flanked and unflanked amplifications at scale, we developed a computational tool, AmpliFinder, which takes short-read sequence data of a bacterial isolate and its reference genome, and identifies and categorizes IS-associated amplification events (Methods and Supplementary Fig. 1). First, a set of IS–chromosome sequence junctions is assembled, including junctions corresponding to IS elements pre-existing in the reference genome, and de novo identified sequence junctions among an IS-side and any chromosomal locus. Then, all non-colocalized IS–chromosome junction pairs are identified (two IS–chromosome junctions matching the two sides of the same IS and mapped to the genome with opposing directions at different loci). Each such pair defines a potential amplicon, whose copy number is then evaluated based on short-read coverage. Finally, the amplification architecture of each potential amplicon is determined by directly testing for the presence or absence of its six type-determining sequence junctions (Methods). This analysis pipeline thereby identifies IS-associated amplifications, their type and copy number and the IS element involved.

## IS-associated amplifications are predominately non-canonical

We applied AmpliFinder to 10,347 isolates (9,696 *Escherichia coli* and 651 *Acinetobacter* *baumannii*) from 48 different sequencing projects of laboratory evolution experiments, both literature curated and new. Growth conditions included selection in benign environments^12,69–73^, nutrient-limiting or starvation conditions^74–77^, selection for phage resistance^19^ and challenges by antibiotics^24,78–84^ or other toxic agents^85–87^ (for a list of experiments, see Supplementary Table 1; for a complete list of isolates, see Supplementary Table 2). The literature curated collection was further enriched with 81 laboratory isolates sampled as part of this study from bacterial cultures previously adapted to the antibiotic chloramphenicol in a morbidostat experiment^24^ as well as 28 isolates from aging bacterial stabs kept at room temperature for an extended period of time (Methods and Supplementary Table 1).

**Fig. 2:**
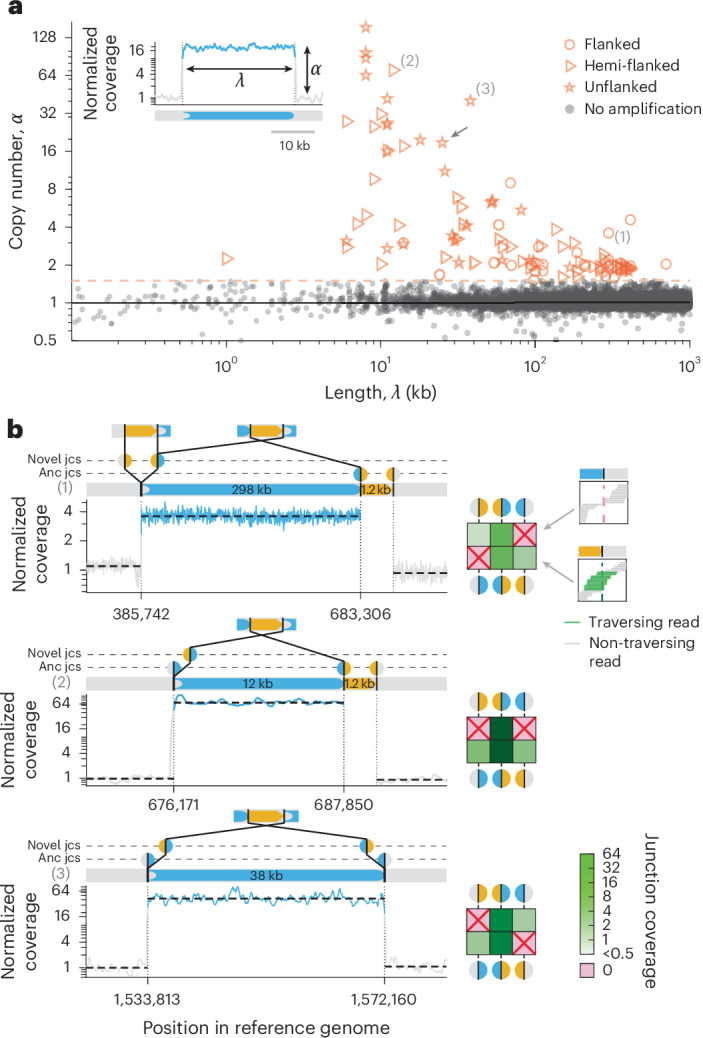
Length and copy number of identified flanked, hemi-flanked and unflanked IS-associated amplifications. **a**, Copy number and length of regions flanked by IS–chromosome junctions of the two sides of the same IS. Amplifications are shown in orange (copy number > 1.5). Symbols indicate amplification types based on coverage analysis of type-determining junctions and visual inspection of the coverage (see examples in **b**). Inset: Example coverage of a representative amplification (indicated by an arrow in the main plot). **b**, Representative flanked (top), hemi-flanked (middle) and unflanked (bottom) amplification structures. Left: Per-base normalized coverage of amplifications (blue) and their genomic context (grey). Median copy numbers are indicated by horizontal dash lines. The schematic above each plot represents the amplicon (blue), periamplicon chromosome (grey) and IS elements (yellow), together with their junctions (circles). For each structure, the four supporting junctions are shown. Ancestral junctions are drawn immediately above the chromosome, while novel junctions, supporting both transposition events and internal IS elements, are drawn above them. Reference IS elements are drawn inline, while de novo transposed IS elements and internal IS elements are drawn above their respective IS–chromosome junctions. IS elements are drawn larger than their relative length, for visibility. Right: Normalized coverage of the six structure-identifying junctions by junction-traversing reads (green shades; Methods). Zero coverage is indicated in pink and a red ‘×’. Blowouts are examples of coverage of an uncovered (top) and covered (bottom) junction showing non-traversing (grey) and traversing (green) reads. Source data

IS-associated amplifications were highly abundant and were dominated not by canonical but rather by non-canonical structures. In *E. coli*, we identified 106 unique amplifications, each characterized by a distinct set of sequence junctions, across the entire dataset (Fig. 2a, Supplementary Table 3 and unresolved structures reported in Supplementary Table 4). Interestingly, of these amplifications, only 25 were identified as IS-flanked canonical amplifications, compared with 81 non-canonical amplifications (54 hemi-flanked and 27 unflanked; Fig. 2a). For the flanked amplifications, 40% of the cases (10/25) involved amplicons that were flanked from both sides by IS elements pre-existing in the ancestral genome, while the remaining (15/25) involved a single de novo transposition (Fig. 2b, top). In the case of hemi-flanked amplifications, some were based on a pre-existing IS on their flanked side (44/54) while the others had a de novo transposition (10/54; Fig. 2b, middle). Lastly, the unflanked amplifications were all identified by two de novo junctions at both sides of the amplicon yet without creating any new junction of the IS and the periamplicon regions (Fig. 2b, bottom). Similarly, in *A. baumannii*, seven unique amplifications were identified, of which six were non-canonical (four hemi-flanked, two unflanked; Supplementary Table 3).

## Long-read validation of canonical and non-canonical structures

**Extended Data Fig. 2:**
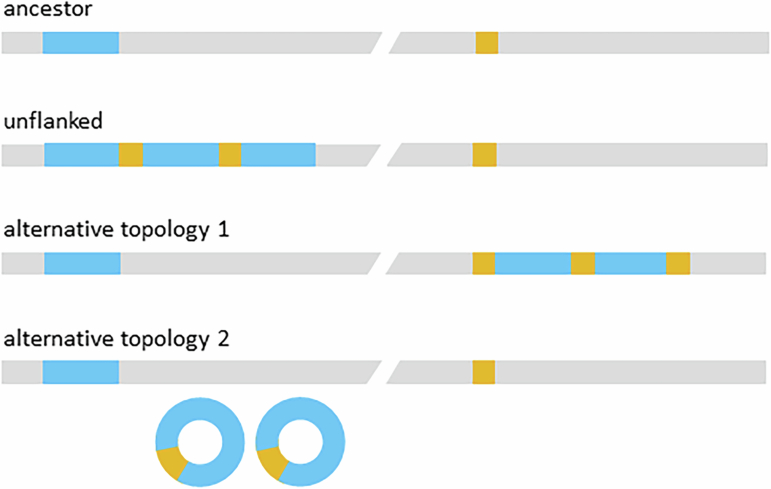
Inferred unflanked amplification structure and putative alternative assemblages. Schematic depicting three alternative putative topologies which are expected to result in a similar set of IS-chromosome junctions, and are therefore indistinguishable by short-read sequencing. The ancestral strain has an IS element at its original locus (yellow) and only a single copy of the amplicon (light blue, top). An unflanked structure has an IS element separating tandemly arrayed amplicons (second from top). In Alternative topology 1, additional copies of the amplified regions are flanked between IS elements at the ancestral locus of the IS element. In Alternative topology 2, amplicons and IS elements form additional circular plasmid-like structures.

**Extended Data Fig. 3:**
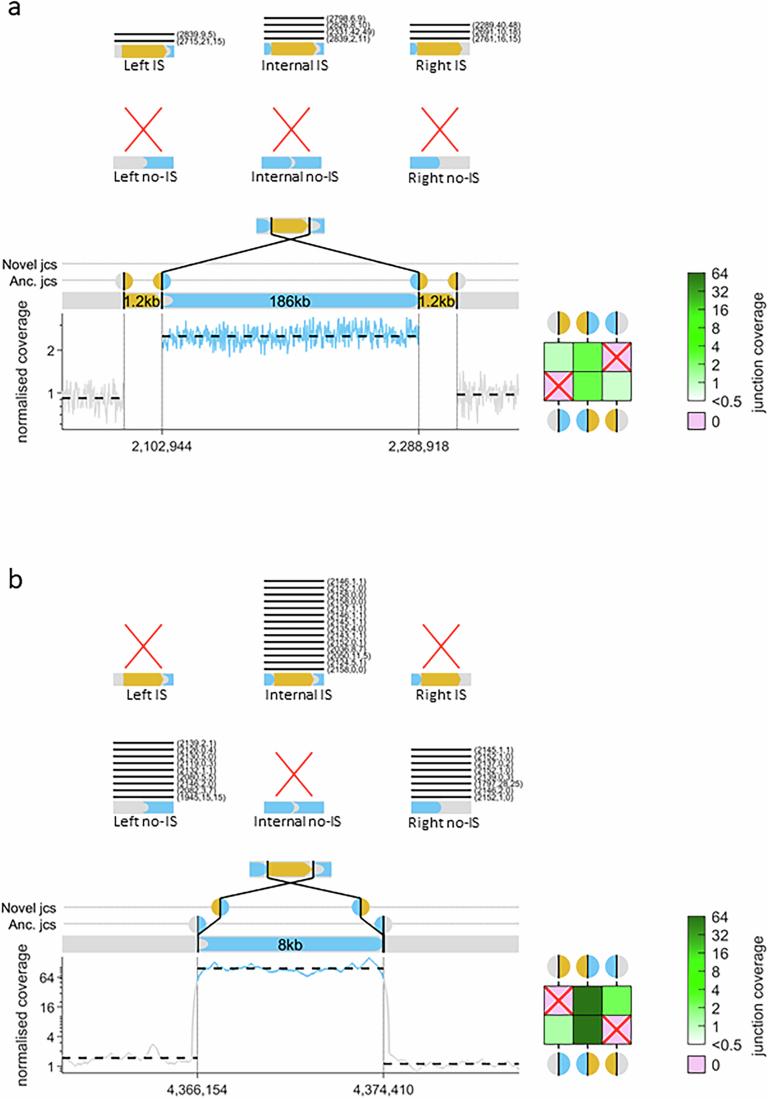
Analysis of long IS-spanning sequencing reads validates canonical flanked and non-canonical unflanked amplification structures. Combined short reads (bottom) and long reads (top) analysis of isolates predicted by AmpliFinder to carry IS-associated amplifications. Short reads analysis indicates copy number of amplifications together with their genomic context (left) and coverage of structure-identifying junctions (right). For long reads analysis, reads are blasted against 6 synthetic junctions: Left IS, internal IS, Right IS, Left no-IS, Internal no-IS and right no-IS. Each junction is either an entire IS element (yellow) with two expected flanks (grey or cyan), or the same genomic junctions lacking an IS between them (no-IS, Methods). Long reads junction analysis requires the read to traverse the entire IS element, thereby positioning it in a 2-sided genomic context. Long reads are depicted as black lines above the junctions with an indication of hit score, distance of alignment start or end from left and right ends of the synthetic junction. A maximum of 14 random reads are presented for each long reads analysis junction. **a**, canonical flanked 186 kb long amplification in isolate SRR21726401. **b**, non-canonical unflanked 8 kb long amplification in isolate SRR23196721. Only 14/317 hits of the internal no-IS synthetic junctions are shown for this isolate. Source data

The observed patterns of copy number changes in conjunction with the presence and absence of the six type-identifying junctions, which we interpreted as the putative non-canonical structures depicted in Fig. 1a, can theoretically be the result of other genomic topologies (Extended Data Fig. 2). Differentiating between these topologies can be done using long reads that either span an entire IS element, thereby determining its position within the amplification array, or ultimately, by identifying ultra-long reads that span the entire amplification array. Starting with canonical amplifications, we applied long-read sequencing to an isolate predicted by AmpliFinder to carry a flanked amplification. Identifying reads traversing the predicted IS elements associated with the amplification structure validated the internal as well as the two flanking ISs (Methods and Extended Data Fig. 3a). Conversely, an isolate predicted by AmpliFinder to carry an unflanked amplification had the internal IS, yet neither of the flanking IS elements (Extended Data Fig. 3b).

To directly resolve the entire structure of amplifications and their dynamics under selection, we applied ultra-long-read sequencing to two clones independently selected for chloramphenicol resistance, one from Toprak et al.^24^ identified by AmpliFinder as carrying a 17,585-bp unflanked IS-associated amplification (Supplementary Table 3), and the other from Lázár et al.^80^, in which AmpliFinder identified a 31,028-bp hemi-flanked IS-associated amplification including a de novo transposition on one side (Supplementary Table 3). Both amplifications surrounded the *mdfA* gene, a dedicated efflux pump with high specificity for chloramphenicol, known as a common evolutionary path for adaptation to the drug^88^.

**Extended Data Fig. 4:**
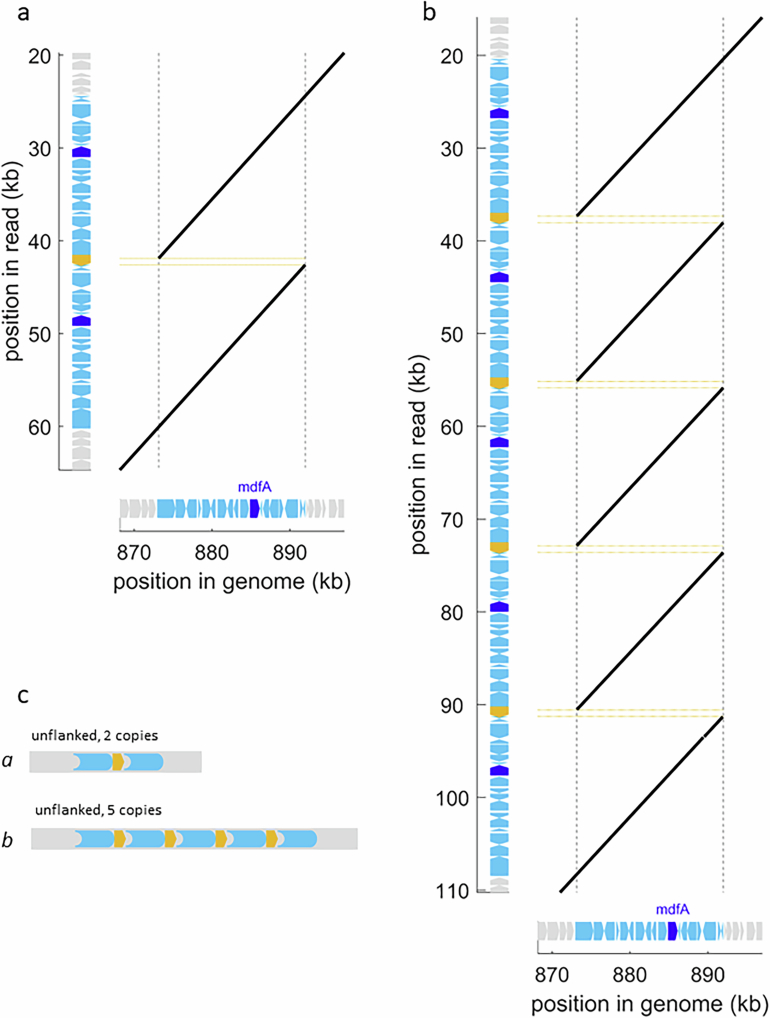
Long-read sequencing captures entire unflanked duplication and amplification structures. **a, b**, Examples of two long reads from cultures of an unflanked duplication carrying isolate (dup^+^) from the morbidostat experiment, as determined by AmpliFinder. The isolate was grown overnight in the absence (**a**) and presence (**b**) of chloramphenicol. Read lengths are 74,537 bases (**a**) and 110,278 bases (**b**). Reads were aligned to the reference genome. A discontinuous alignment covers the amplicon (coloured) twice (**a**) and 5 times (**b**), indicating a duplication and amplification. In both cases, all copies were separated by the same IS element (yellow). Amplified genes are indicated in light blue on the X and Y axes, and the chloramphenicol resistance-conferring gene *mdfA* borne by the amplicon is indicated in dark blue. **c**, cartoon representation of the two amplicon arrays identified by long reads in (**a**) and (**b**). Source data

**Extended Data Fig. 5:**
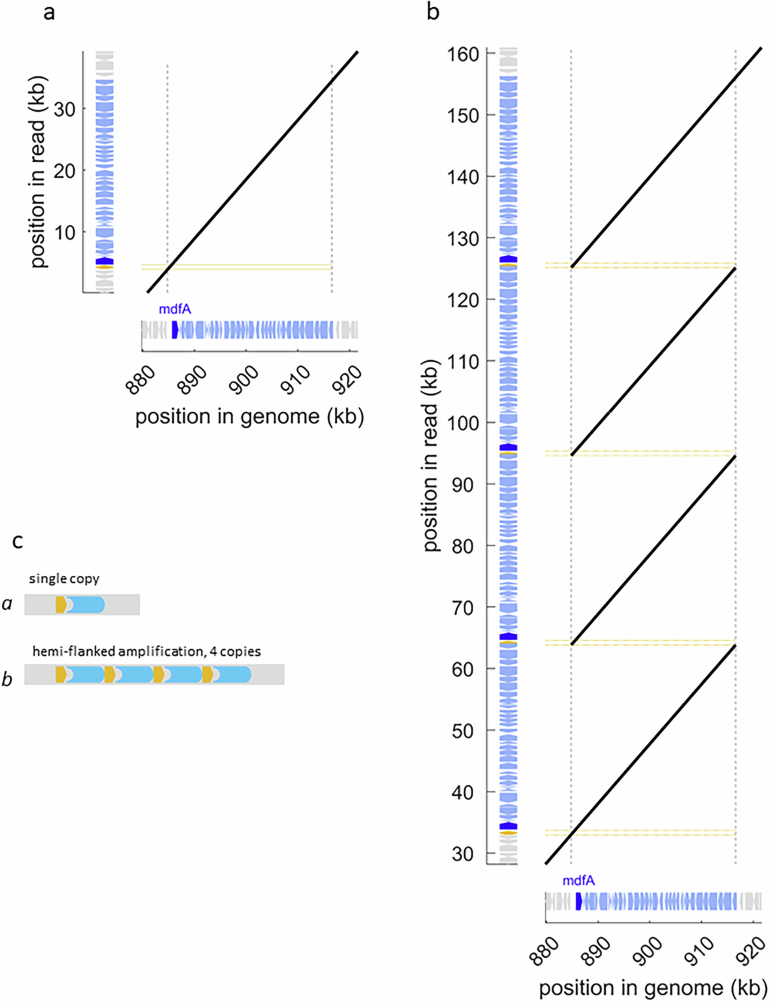
Long-read sequencing captures non-canonical hemi-flanked amplification structure. **a, b**, Examples of two long reads from cultures of the same CHL-9 isolate from laboratory evolution experiment by Lázár et al. (see Extended Data Table 1). The isolate was grown overnight in the absence (**a**) and presence (**b**) of chloramphenicol. Read lengths are 64,334 bases (**a**) and 200,294 bases (**b**). Reads were aligned to the reference genome. A discontinuous alignment covers the amplicon (coloured) once (**a**) and 4 times (**b**), indicating a single copy and an amplification. The single copy is bordered by an IS element from one side and all copies are separated by the same IS element (yellow), indicating a IS-associated hemi-flanked amplification. Amplified genes indicated in violet on X and Y axes, and the chloramphenicol resistance-conferring gene *mdfA* borne by the amplicon is indicated in dark blue. **c**, cartoon representation of the two amplicon arrays identified by long reads in (**a**) and (**b**). Source data

Analysing long reads from cultures grown in the absence and presence of chloramphenicol selection confirmed the non-canonical structures and revealed their reversible nature. We cultured both the hemi-flanked and unflanked *mdfA-*amplified clones and extracted their DNA for ultra-long-read sequencing (Methods). For cultures grown in the presence of the antibiotic, alignment to the reference genome of long reads whose ends match the two chromosomal regions bordering the amplicon, identified the AmpliFinder-predicted IS-associated amplification structures. In both cases, IS elements separated multiple consecutive copies of the amplicon. For the predicted hemi-flanked amplification, the array was indeed flanked from one side only, and for the predicted unflanked amplification, no flanking IS elements were observed (Extended Data Figs. 4b and 5b). For cultures grown without antibiotics, reads traversing the entire amplification array identified only duplications or single-copy structures (Extended Data Figs. 4a and 5a). These observations confirm the predicted non-canonical architectures, highlight their rapid dynamics and show that they can be readily reduced, probably by homologous recombination, to a single copy lacking any amplification-facilitating homology.

## Non-canonical unflanked amplifications are shorter and have a higher copy number

**Fig. 3:**
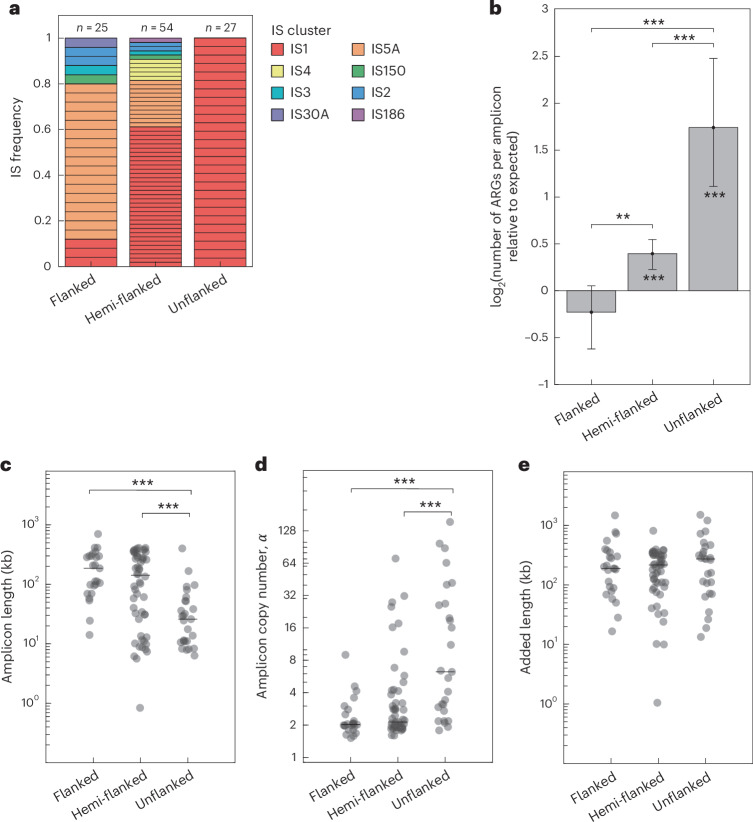
Non-canonical amplifications are uniquely associated with IS1 elements and more specifically and efficiently amplify genes under selection. **a**, IS element sequence clusters identified across amplification structures (Methods). IS1 is enriched in both hemi-flanked and unflanked amplifications (*P* value: 2 × 10^−3^ and 2 × 10^−10^, respectively, Bonferroni corrected, two-sided Fisher’s exact test; Methods). **b**, Non-canonical amplifications are enriched for genes under selection. Amplifications observed in adaptive laboratory evolution experiments in which adaptation was towards antibiotic drugs were enriched for ARGs compared with the expected (fraction of the bootstrap rate below expected, *P* value: 4 × 10^−4^ for hemi-flanked, <1 × 10^−4^ for unflanked; Methods). Non-canonical amplifications were enriched for genes under selection compared with canonical, flanked (*n* = 14), amplifications, and unflanked amplifications (*n* = 14) were enriched compared with hemi-flanked (*n* = 36) amplifications (fraction of permutations in which difference is larger than observed; Methods). Error bars indicate the 5th–95th percentile of 10,000 bootstrap resamples of amplicons. **c**, Unflanked amplifications (*n* = 27) are shorter than either hemi-flanked (*n* = 54) or flanked (*n* = 25) amplifications (*P* value = 5 × 10^−6^ and 4 × 10^−3^, Bonferroni-corrected Mann–Whitney *U* test). **d**, Unflanked amplifications (*n* = 27) have higher copy numbers than either the hemi-flanked (*n* = 54) or flanked (*n* = 25) amplifications (*P* value = 4.1 × 10^−4^ and 4.3 × 10^−5^, Bonferroni-corrected Mann–Whitney *U* test). **e**, The number of bases added to the bacterial chromosome by amplifications across different amplification types, calculated as amplicon length times the number of added amplicon copies. Unflanked (*n* = 27), hemi-flanked (*n* = 54) and flanked (*n* = 25) amplifications. Source data

We next asked whether the IS elements found in non-canonical amplifications differ from IS elements found in canonical ones. Clustering the ISs associated with each of the 106 amplifications identified in *E. coli* by sequence homology (Methods), we identified 8 distinct types of ISs over all amplification types. Notably, these IS types were not randomly distributed among the flanked, hemi-flanked and unflanked amplifications: compared with the IS-flanked amplification structures, which were from six different IS types (Fig. 3a), the hemi-flanked amplification structures were dominated by IS1, and quite strikingly, the unflanked amplifications were exclusively of this IS1 type (*P* values of 2 × 10^−3^ and 2 × 10^−10^, respectively, Fisher exact, Bonferroni corrected; Fig. 3a).

**Extended Data Fig. 6:**
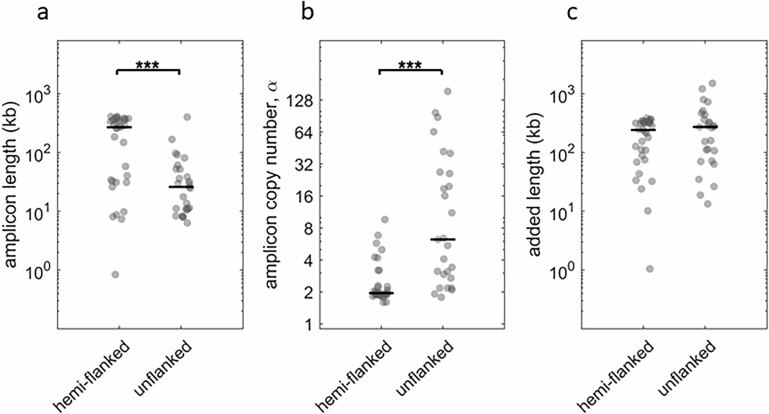
IS1-associated unflanked amplifications are shorter and reach a higher copy number than IS1-associated hemi-flanked amplifications. **a**, Unflanked amplifications (n = 25) are shorter than hemi-flanked (n = 33) amplifications (p^−^ value = 9 × 10^−6^, Mann-Whitney U test). **b**, Unflanked amplifications (n = 25) have higher copy number than hemi-flanked (n = 33) amplifications (p^−^ value = 5 × 10^−4^, Mann-Whitney U test). **c**, The number of bases added to the bacterial chromosome by different amplification types, calculated as amplicon length times the number of added amplicon copies. Unflanked (n = 25) and hemi-flanked (n = 33) amplifications. Source data

Inspired by the observation that non-canonical amplifications are generated by specialized IS elements, we hypothesized that non-canonical amplifications may also differ from canonical amplifications in copy number and length. Comparing the copy number of IS-associated amplifications of different types, we found that unflanked amplifications were of significantly higher copy number, with a median of 6.2 compared with 2.0 and 2.1 for flanked and hemi-flanked amplifications, respectively (*P* value = 4.3 × 10^−5^ and 4.1 × 10^−4^, Bonferroni-corrected Mann–Whitney *U* test; Fig. 3d). Analysing the amplicon length, we found a complementary trend: unflanked amplifications were much shorter, with a median amplicon length of 26 kb compared with 186 kb and 142 kb for flanked and hemi-flanked (*P* value = 5 × 10^−6^ and 4 × 10^−3^, respectively; Bonferroni-corrected Mann–Whitney *U* test; Fig. 3c). Overall, the length and copy number were inversely related (*R* = −0.64, *P* value < 10^−10^) and the number of additional base pairs due to each amplification, defined as the product of the amplification length and the number of added copies, showed no significant difference between amplification types (Fig. 3e). These differences in amplicon copy number and length were observed also when comparing unflanked and hemi-flanked amplifications associated with IS1 only, suggesting that the difference between amplification types is not explained by the difference in IS elements alone (Extended Data Fig. 6). These results suggest that while the cost of amplification due to additional bases is similar for different types of amplification, unflanked amplicons are often more efficient as their shorter length allows them to reach higher copy numbers, presumably allowing a larger increase in the expression of enclosed genes^89^.

Finally, lacking flanking IS elements, which otherwise disrupt the ancestral amplicon–periamplicon sequence, non-canonical amplifications are hypothesized to be less restricted in their locations. In particular, we asked whether it is possible for the non-flanked ends of non-canonical amplifications to lie even within essential genes. We identified essential genes in each reference genome based on homology to the list of essential genes^90^, and determined for each end of each de novo amplification whether it lies within these mapped essential genes. Our analysis identified three examples of non-flanked amplification ends that lie within the open reading frames of essential genes (*aspS*, *spoT* and *leuS*). As expected, we did not identify any IS-flanked side that mapped into essential genes. The possibility of creating amplicons whose sides are unrestricted may contribute to the observed effectiveness of non-canonical amplifications in amplifying targeted short regions.

## Non-canonical amplifications are enriched for genes under selection

It is often assumed that gene amplifications deliver a fitness advantage by increasing the gene dosage of specific genes under selection. Given their shorter lengths, we wondered whether non-canonical unflanked IS-associated amplifications are still able to capture specific genes under selection. Focusing on the subset of laboratory experiments in which *E. coli* were adapting to antibiotic drugs (Supplementary Table 1), we counted the total number of antibiotic-resistance genes (ARGs) enclosed within all identified flanked, hemi-flanked and unflanked amplicons. To quantify enrichment, we normalized these ARG counts to the average number observed when the locations of these same amplicon lengths are randomly chosen in the reference genome (Supplementary Table 5). The non-canonical, hemi-flanked and unflanked amplifications had 1.3 and 3.4 times more resistance genes than expected by chance, in contrast to the canonical amplifications, which did not show a significant enrichment (Fig. 3b; *P* values of 4 × 10^−4^, <1 × 10^−4^ and 0.91, respectively; bootstrapping; Methods). Comparing enrichment levels between amplification types, we found that unflanked were significantly more enriched in ARGs than either hemi-flanked or flanked (*P* values: <1 × 10^−4^ and <1 × 10^−4^ respectively; permutation test). The non-canonical unflanked amplifications therefore most effectively enclose genes under selection. In addition to ARGs, multiple other genes important for adaptation to various environmental stresses were identified in non-canonical amplifications. In two amplicons identified in isolates collected from the MEGA-plate^82,91^, we identified *cheA*, which is expected to be under selection in these chemotactic settings. Similarly, in amplicons detected in isolates from long-term adaptation to stationary phase (Supplementary Table 3)^76^, we identified genes encoding multiple glutamate transporters (*abgT*, *gltI*, *gltJ*, *gltJ* and *gltK*) and glutamate metabolism genes (*abgA*, *abgB*) as well as a global stress regulator (*uspE*), all consistent both with the known evolutionary pathways to adapt to starvation and with SNPs identified in the same study^76^.

## Non-canonical duplications facilitate evolvability

**Fig. 4:**
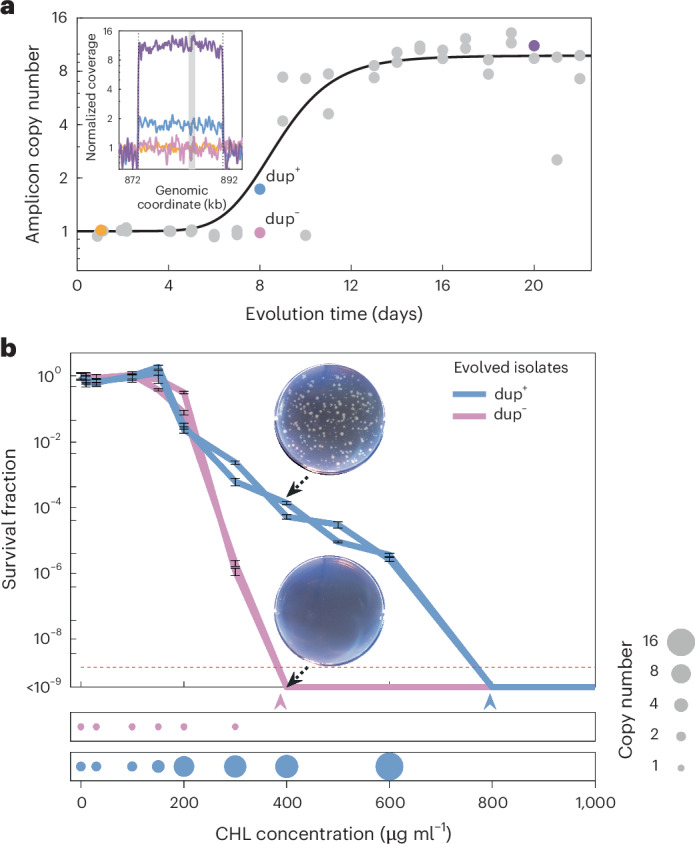
Non-canonical unflanked duplications provide a stepping stone for evolutionary adaptation. **a**, Copy number of a 17,585-bp-long non-canonical, unflanked, amplicon containing the chloramphenicol resistance gene *mdfA* (grey shade in the inset), for isolates from a daily-frozen stock of the morbidostat chloramphenicol adaptation experiment (Methods)^23^. Inset: Per-base normalized coverage of four example isolates (corresponding to coloured dots in the main axis). Vertical dotted lines indicate the boundaries of the amplicon. The grey box indicates the *mdfA* locus. **b**, Two isolates (dup^+^ and dup^−^) taken from day 8 with similar resistance have different evolvability potential. While both isolates have a reduced survival rate starting at 200 μg ml^−1^ chloramphenicol, dup^+^ (blue) has a higher mutant prevention concentration of 800 μg ml^−1^, compared with only 400 μg ml^−1^ for dup^−^ (magenta). The dashed orange line represents the detection limit. Error bars indicate Poisson error on the number of counts. Bottom: The copy number of the *mdfA*-containing amplicon was higher and increased with antibiotic concentration for mutants arising from the dup^+^ isolate, while no change in copy number was observed for resistant mutants arising from the dup^−^ isolate. The horizontal lines represent the means of measurements per amplification type. CHL, chloramphenicol. Source data

**Extended Data Fig. 7:**
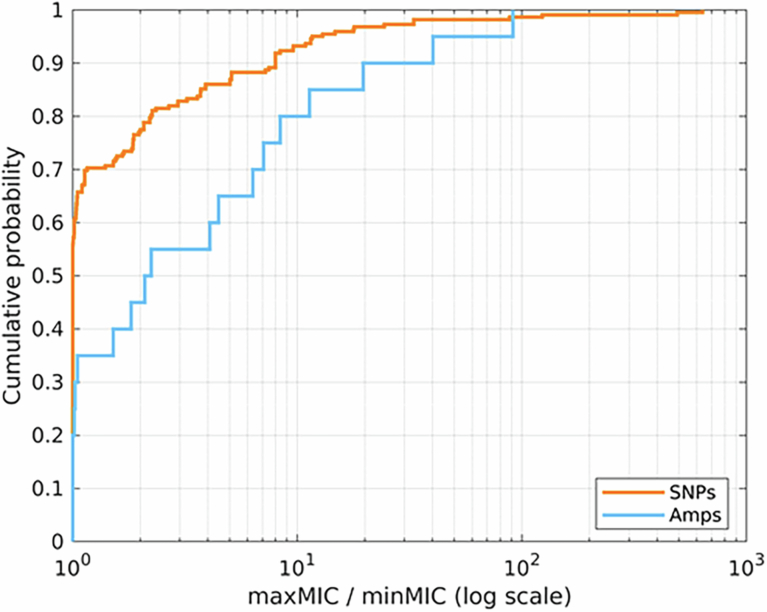
Amplifications are more evolvable than SNPs. Cumulative distribution of the ratios between maximal and minimal MIC values associated with either SNPs (n = 260, orange) or amplifications (n = 16, blue), demonstrating the range of resistance evolution observed per genomic change (Methods). Amplifications had a wider range indicating higher evolvability (KS-test, p^−^value = 0.007).

Once two copies are created, amplification can be driven by homologous recombination, suggesting that a duplication of a gene under selection in a given environment provides increased ability to further adapt to this environmental challenge^3^. Focusing on the morbidostat experiment, in which bacteria were adapting to increasing levels of chloramphenicol^24^, we identified an unflanked amplification structure enclosing the chloramphenicol resistance gene *mdfA* (amplicon 873,161–890,745 in the U00096 GenBank reference; Supplementary Table 3). Whole-genome sequencing isolates from daily-frozen samples, we quantified the copy number of this region during adaptation. The copy number was 1 until day 8, when it started increasing, reaching as high as 13.2 copies by day 20 (Fig. 4a). We focused on two isolates from day 8, one with a non-canonical unflanked duplication (dup^+^) and one without a duplication (dup^−^, copy numbers of 1.7 and 1, respectively). SNP-wise, the only difference between these isolates was a T>G at −34 from the *mdfA* transcription start site suggesting a promoter mutation in dup^−^. To measure the difference in resistance and evolvability of resistance, dense cultures of each of the isolates were plated on a gradient of chloramphenicol and the fraction of resistant mutants was calculated for each antibiotic concentration. We found that although both isolates shared a drop in the surviving fraction at a similar antibiotic concentration, indicating a similar minimal inhibitory concentration (MIC, 200 μg ml^−1^ for both), dup^+^ had a much larger mutant selection window^92^ (MSW, 200–800 μg ml^−1^, compared with 200–400 μg ml^−1^ for dup^−^; Fig. 4b). To test whether these evolved descendents of dup^+^ adapted via amplification of the unflanked duplication structure, we collected isolates from these plates, whole-genome sequenced them and applied AmpliFinder. We found that all isolates that were descendants of the dup^+^ isolate carried the same unflanked structure, with copy numbers of the amplicon increasing with the level of the antibiotic (Fig. 4b). By contrast, descendants of the dup^−^ isolate all carried a single copy of the region (Fig. 4b). In a separate analysis, quantifying the adaptive potential of mutants arising during adaptation to ampicillin^91^, we found that non-canonical amplifications were observed over a wider range of MICs than SNPs, suggesting increased evolvability (Kolmogorov–Smirnov test, *P* = value 0.007; Methods and Extended Data Fig. 7). Together, these results generalize our understanding of amplification as a stepping stone of adaptation to the shorter, more gene-focused, non-canonical amplifications^3^.

## Unflanked amplifications may form via successive replicative transposition events

**Extended Data Fig. 8:**
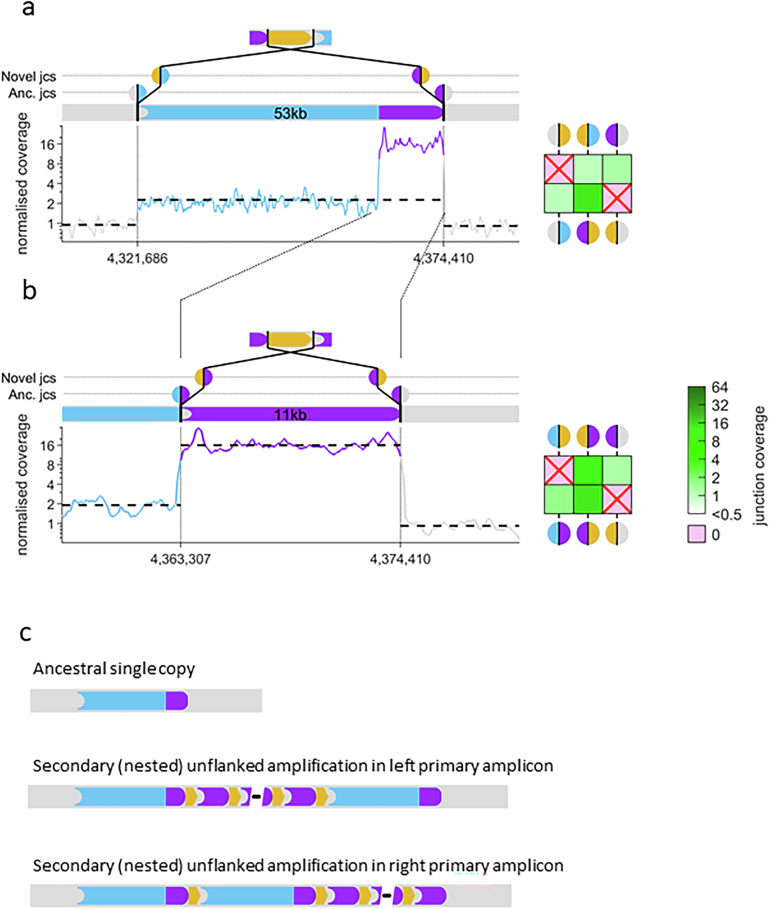
Nested amplification identified by short-read sequencing data. Copy number of amplifications together with their genomic context (left) and coverage of structure-identifying junctions (right) for nested amplifications identified in the same evolved isolate. **a**, an unflanked amplification structure with 2.1 copies is identified between reference positions 4,321,687–4,374,410. **b**, an amplification structure with 16.4 copies is identified between reference positions 4,363,308–4,374,410. GenBank CP009273 was used as reference. **c**, schematic representation of the ancestral structure (top) and two inferred structures in which the nested amplification is either part of the left or right copy of the amplicon (middle and bottom, respectively). Source data

**Extended Data Fig. 9:**
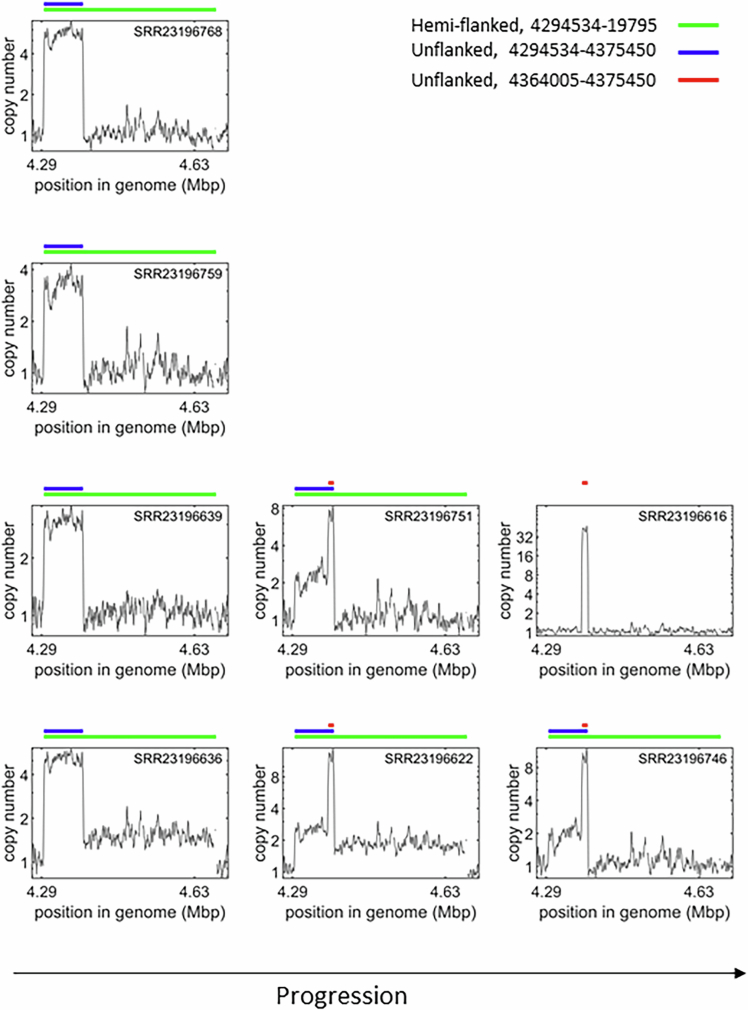
Step-wise formation of a non-canonical unflanked amplification via an intermediate nested structure. Normalized short-read genome coverage of all isolates (n = 8) from sequencing project PRJNA926794 identified by AmpliFinder IS-to-chromosome junction analysis to carry one or more amplicons from the same nested-amplicon group. Plots are organized by the progression of respective isolates along the experimental setup. While early amplicon-carrying isolates (left) have a hemi-flanked amplicon enclosing an unflanked one (green and blue, respectively), intermediate isolates (middle) carry also an additional unflanked amplification structure (red) and in one instance, of a much later isolate (right), this unflanked amplification reaches a much higher copy number while the enclosing amplicons are reversed and not observed, by either coverage analysis or the type-determining sequence junctions. Green, blue and red bars mark the span of the amplicons and are drawn for isolates in which required IS-junctions are identified. Amplicon coordinates in genome (NCBI CP009273) are shown in the legend. Genome length is 4,631,469. Source data

**Extended Data Fig. 10:**
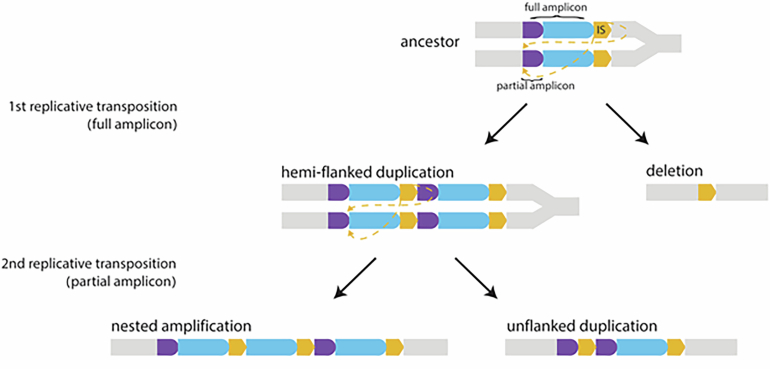
Suggested model for the formation of non-canonical unflanked amplifications by successive replicative transpositions. An IS element in a replicated chromosome undergoes replicative transposition to a site on the second copy of the chromosome (top, see Extended Data Fig. 1 for detailed reaction). The region between the ancestral IS site and the novel integration site is designated full amplicon (cyan and purple combined). This replicative transposition results in two products, a hemi-flanked duplication of the full amplicon (middle left) and a reciprocal deletion (middle right). The duplication then undergoes a subsequent similar replicative transposition step, but of a shorter partial amplicon (purple) resulting in a nested amplification product (left) and in an unflanked amplification (right).

While hemi-flanked amplifications may be created through a replicative transposition-based mechanism (Extended Data Fig. 1)^35^, how unflanked amplifications are formed remains unclear^54,59–61^. Integrating same-isolate amplification events, we identified multiple instances of nested amplifications, in all of which the internal amplification was unflanked and always shared one of its ends with the external enclosing amplification (Supplementary Table 6 and Extended Data Fig. 8). Furthermore, analysing lineages of evolved isolates, we identified a nested unflanked amplification observed in early isolates, which was then followed by a later isolate carrying the same exact unflanked amplification, yet with no trace of the enclosing amplicon (Extended Data Fig. 10). On the basis of these observations, we suggest that nested amplifications could be intermediate forms in a series of two successive replicative transpositions, leading to the formation of unflanked amplifications (Extended Data Fig. 9). This model for the creation of unflanked amplifications is further consistent with the shorter length of unflanked amplifications and with the strong enrichment towards a specific IS element in non-canonical amplifications, presumably reflecting the replicative transposition capacity of this IS1 (Extended Data Figs. 1 and 9)^55^.

## Discussion

Analysing a large set of public and newly created sequencing data of ancestral and lab-evolved *E. coli* and *A. baumannii* strains, we unravel an important mode of adaptive evolution through non-canonical IS-mediated gene amplifications. We found that these non-canonical amplifications, in which the IS is only flanking the amplified array from one side, or even not at all, are in fact more common than the classically expected IS-flanked amplifications. Such non-canonical amplifications more effectively amplify genes under selection: they are shorter and reach a higher copy number, and the position of their ends is unrestricted and can even lie within essential genes. Long-read sequencing confirms these structures and shows that following removal of selection, these structures can contract to single copies without any scars at the amplified locus. Lastly, measuring the evolvability of resistance, we show how such non-canonical duplications provide an evolutionary stepping stone for rapid adaptive evolution via expansion and contraction of the amplification array.

Our results hint at a plausible model for the formation of unflanked amplifications. Two successive replicative transpositions acting between sister chromatids may result in an unflanked duplication. This model is supported by our observation of unflanked amplifications often being identified enclosed within other amplifications, which are also formed by similar successive replicative transposition steps, and by our observation of such a nested structure preceding a later fixated architecture in which the same unflanked amplification appears un-nested. In principle, unflanked amplifications could also form by post-duplication excision of flanking IS elements, although such a mechanism is not consistent with the lack of junction scars in our data, with the observed retention of internal IS elements, with the observed shorter lengths of unflanked amplifications and with the observation of amplicon borders overlapping essential genes. While the two-step replicative transposition model we propose is consistent with these observations, further studies are needed to fully validate this model and understand its preference for specific IS elements and amplicon lengths.

Our study has several limitations. Our analysis is based on *E. coli* and *A. baumannii* laboratory-evolved strains with most of the data coming from *E. coli* strains. The mechanisms underlying the non-canonical IS-mediated amplifications we describe might show species-specific variations that are not captured in this work. Our work is further based on bacterial isolates from lab evolution experiments, and it is possible that such conditions may bias towards specific modes of IS-associated amplification, which may differ from those common in natural environments or in the clinic. Our computational tool, AmpliFinder, relies on a reference genome, and correct identification of an amplicon is dependent upon its intact presence in this reference. Direct validation of structures predicted by AmpliFinder was done only for representative isolates with long-read sequencing, and it is theoretically possible that some amplifications may have a different underlying topology. We further note that resolving complex topologies such as nested amplifications requires manual inspection of amplicon coverage. Furthermore, as the copy number determined by short-read coverage represents the population average, our analysis cannot resolve within-sample variations and may even miss detection of amplifications carried by a fraction of the population. Finally, our analysis of genomic constraints on amplification edges is based on a database of essential genes while gene essentiality may vary under different experimental conditions^93^.

The abundance of non-canonical amplifications identified in this work has major implications. As ancestral sequences are not disrupted at unflanked edges of an amplicon, non-canonical amplifications are less constrained, allowing more targeted and focused amplification of specific genes under selection. Furthermore, as ancestral amplicon ends are retained, these non-canonical structures can scarlessly reduce back to a single copy. Finally, identifying IS1 as a major player underlying non-canonical unflanked amplifications, our findings point to the potential of IS-targeting evolution-inhibiting treatment, which is expected to reduce the rate of microbial adaptation to antibiotic treatments.

## Methods

### Sequencing evolved bacterial isolates

Isolates were collected from two different sources: frozen stocks of the morbidostat experiment^24^ and aging bacteria from 10-year-old agar stabs (Supplementary Table 1). For the morbidostat experiment, in which bacteria grew under selective pressure of chloramphenicol^24^, daily samples kept frozen in 15% glycerol at −80 °C were thawed and spread on LB agar plates. For the bacterial agar stabs, kept sealed in a screw cap tube for >10 years, samples were spread on SOC agar plates. For both experiments, individual colonies were collected after overnight incubation at 37 °C and cultured at 37 °C in liquid LB for 9 h. DNA was extracted using the Roche High Pure PCR Template Preparation Kit. DNA concentration was measured by Quant-iT (Thermo Fisher Scientific). Illumina sequencing libraries were prepared as previously described^94^. Briefly, 1.5 ng DNA was tagmented (Illumina, FC-121-1030). Tagmented libraries were indexed and amplified by the KAPA HiFi HotStart kit (Roche, KK2611). Size selection and clean-up of libraries were done using 0.8 volumes of AMPure beads (Beckman Coulter). Libraries were sequenced in rapid mode on an Illumina HiSeq 2500 to produce paired-end reads.

### Whole-genome short-read sequencing database curation and filtering

Short-read sequencing data of 10,347 ancestral and evolved isolates were curated from 45 different laboratory evolution studies (Supplementary Table 2). For each sequencing project (NCBI BioProject), all associated read files were downloaded in *fastq* format (fasterq-dump version 3.0.0). Read length was determined by randomly sampling 100 reads from each file. Entries with nonuniform read length (maximal length difference between sampled reads exceeds 5%) underwent read trimming and/or filtering by minimal length using fastp^95^ version 0.23.4. For entries with a cumulative read length exceeding 5 × 10^8^ bases, reads were randomly downsampled to 5 × 10^8^ using seqkit^95,96^ version 2.3.0 to reduce use of downstream processing resources. Isolates were labelled as either ancestral or evolved based on published references or Sequence Read Archive (SRA) comments, and each evolved isolate was assigned an ancestral one for downstream analysis. Assigned ancestral isolates were preferably (and mostly) from the same sequencing project. If none was available, an ancestor from a different sequencing project, which is expected to be derived from the same reference strain and which was sequenced with a similar read length, was assigned. Finally, each isolate was also assigned a reference genome that best represents the specific strain used in the experiment based on strain identity as mentioned in the published reference paper. Evolved isolates and their ancestors always share a reference genome. All isolates and their assigned ancestor and reference are listed in Supplementary Table 2.

### Identifying IS-associated amplifications using short-read sequencing (AmpliFinder)

AmpliFinder takes an input of short reads of two bacterial isolates: evolved and ancestor, in the form of fastq files, as well as a reference genome, and outputs IS-associated amplifications including the amplified region, the copy number, the involved IS and the inferred genomic structure (flanked, hemi-flanked and unflanked). It includes the following steps:

#### Identifying IS elements in reference genomes

For each reference genome, IS loci are identified based on GenBank annotation, when available. For bacterial reference genomes lacking IS element annotation, IS elements are identified using BLAST (version 2.10.1) of the bacterial genome sequence against the ISfinder database^97^ (retrieved October 2020; filtering for alignment hits with a length of at least 0.9 of the annotated IS element). For each IS element *i*, we denote its genomic position by the position of its left and right sides indicated as ${\mathrm{IS}}_{i}^{{\rm{L}}},\,{\mathrm{IS}}_{i}^{{\rm{R}}}$. AmpliFinder also allows identifying IS elements in the reference genome by implementing ISEScan^98^, but this is not used in the analysis done as part of this study.

#### Identifying ancestral and de novo IS–chromosome junctions

For each isolate, short reads are aligned to the corresponding reference genome using breseq version 0.32.1 (refs. ^65,66^), with default parameters. Predicted sequence junctions (points where two nucleotide sequences that exist separately in the reference genome are joined together) are extracted from the breseq output file (output.gd). Both sides of each junction are then compared with the sequences of all IS elements found in the reference genome. In this comparison, the IS element is considered together with an additional 100 bp from the bacterial genome on each side. A junction side is assigned as an IS side ${\mathrm{IS}}_{i}^{\mathrm{side}}$ (*i* is the index of the IS element, side refers to L or R) if it fully matches the IS reference sequence starting at a distance of up to 10 bp from the edge of the IS element. Such an ‘IS side’ of a junction may match one or more IS sides (matching multiple ISs is common because of high homology among different copies of a given IS element in the reference genome). A side not assigned any IS is considered a ‘chromosome side’. We consider a junction to be an IS–chromosome junction if one of its two sides is an ‘IS side’, matching the side of at least one IS, and the other is a ‘chromosome side’, matching a unique non-IS locus in the genome. Each such IS–chromosome junction is denoted as ${\mathrm{IS}}_{i}^{\mathrm{side}}\leftrightarrow {C}_{p}^{\mathrm{dir}}$, where ${\mathrm{IS}}_{i}^{\mathrm{side}}$ denotes the specific IS and the side matched by one side of the junction, and ${C}_{p}^{\mathrm{dir}}$ denotes the genomic position *p* and direction, dir = +/− of the match (if the junction’s IS side matches more than one reference IS side, we consider all matching IS elements as candidates). In addition to the de novo IS–chromosome junctions, we also list all the native IS–chromosome junctions of the reference genome.

#### Identifying IS-associated transpositions and potential amplifications

Starting with the set of IS–chromosome junctions created above, we identify all pairs of these junctions whose IS side matches two different sides of the same reference IS and whose chromosome sides match at opposite directions on the chromosome (at any two, not necessarily colocalized, loci), namely, ${J}_{i,\,{p}_{+}{,p}_{-}}^{\to}=({\mathrm{IS}}_{i}^{{\rm{L}}}\leftrightarrow {C}_{{p}_{-}}^{-},\,{\mathrm{IS}}_{i}^{{\rm{R}}}\leftrightarrow {C}_{{p}_{+}}^{+})$ or ${J}_{i,\,{p}_{+}{,p}_{-}}^{\leftarrow }=({\mathrm{IS}}_{i}^{{\rm{R}}}\leftrightarrow {C}_{{p}_{-}}^{-},\,{\mathrm{IS}}_{i}^{{\rm{L}}}\leftrightarrow {C}_{{p}_{+}}^{+})$. Junction pairs in which the two sides of the IS map to the same or very close position in the chromosome, $\left|{p}_{+}-{p}_{-}\right| < 30$, are considered de novo transpositions. All other junction pairs are considered ‘potential amplifications’, with an amplicon extending from ${p}_{+}$ to ${p}_{-}$ and a length $l={p}_{-}-{p}_{+}$ (or $l=L+{p}_{-}-{p}_{+}$ if ${p}_{-} < {p}_{+}$, where L is the length of a circular reference genome). Potential amplicons with length *l* > 1 Mb were discarded from downstream analysis.

#### Calculating copy numbers of potential IS-associated amplifications

For each potential amplicon, the read coverage of each base through the amplicon, as determined by breseq version 0.32.1 and written as a standard breseq output in <ref>.coverage.tab file, was normalized by the median genome coverage of the isolate to give a self-normalized per-base copy number. These self-normalized per-base copy numbers were then normalized by the self-normalized per-base copy number of the ancestor, yielding an ancestor-normalized per-base copy number. The amplicon coverage was calculated as the median ancestor-normalized per-base copy number across the amplicon. Any potential amplification with coverage >1.5 was considered an amplification.

#### Scoring presence and absence of the six type-determining junctions

For each amplification, defined by the junction pair ${J}_{i,\,{p}_{+}{,{p}}_{-}}^{\to }$ or ${J}_{i,\,{p}_{+}{,{p}}_{-}}^{\leftarrow }$, we synthetically constructed the following six junction sequences (for simplicity, shown only for ${J}_{i,\,{p}_{+}{,{p}}_{-}}^{\to }$): two IS–amplicon junctions (${\mathrm{IS}}_{i}^{{\rm{L}}}\leftrightarrow {C}_{{p}_{-}}^{-}$ and $\,{\mathrm{IS}}_{i}^{{\rm{R}}}\leftrightarrow {C}_{{p}_{+}}^{+}$), two IS–periamplicon junctions (${\mathrm{IS}}_{i}^{{\rm{L}}}\leftrightarrow {C}_{{p}_{+}}^{-}$ and $\,{\mathrm{IS}}_{i}^{{\rm{R}}}\leftrightarrow {C}_{{p}_{-}}^{+}$) and the two ancestral periamplicon–amplicon junctions (${C}_{{p}_{-}}^{+}\leftrightarrow {C}_{{p}_{-}}^{-}$ and $\,{C}_{{p}_{+}}^{-}\leftrightarrow {C}_{{p}_{+}}^{+}$). These synthetic sequence junctions represent the different options in which the sequences of the predicted amplicon, the region bordering it from both sides (‘periamplicon’) and the implied IS, are expected to be joined in different amplification structures. In cases when a matching IS element is found at any side of an amplification, either identified in the reference genome or as a de novo transposition, the position of the periamplicon region was extracted from the reference genome or the de novo identified corresponding junction. Each junction was constructed by concatenating 2-read length of its two corresponding reference genome regions, creating a 4-read length sequence. The short-read data of the isolate were aligned to these 6 junction sequences, and the number of junction-supporting reads for each junction was defined as the number of reads traversing the junction (with at least 12 bases on each of its sides). A minimum number of five traversing reads was required to make a call. Junction coverage in copy number units was calculated as the number of junction-supporting reads divided by the expected number of these reads per single copy, which was estimated as *μ*(*λ* − 2*Ω* + 1)/*λ*, where *μ* is the median number of reads aligned to covered loci in the reference genome, *λ* is the length of the aligned reads and *Ω* is the minimal overlap of the read with each side of the junction required to be considered a junction-supporting read. Amplifications were further verified by visually inspecting the coverage of the amplicon and the surrounding region in the chromosome as well as the coverage of the expected junctions.

### Identifying nested amplification groups

Nested amplifications were identified within the set of visually inspected non-canonical amplifications. Pairwise comparison of coordinates identified candidates: amplifications fully contained within each other. These were then visually inspected in all isolates in which they were algorithmically identified by AmpliFinder. Only overlapping amplifications found in the same isolate were considered nested. These were later probed for shared borders.

### Whole-genome ultra-long-read sequencing

We applied ultra-long-read sequencing to bacterial samples expected to carry either hemi-flanked or unflanked amplification according to AmpliFinder analysis. This sequencing was applied to directly observe the predicted structure. An *E. coli* overnight culture was centrifuged at 3,220*g* for 10 min, and the supernatant was decanted. The pellet was resuspended by pipette mixing in 100 µl sterile phosphate buffer solution followed by the addition of 10 ml of lysis buffer (10 mM Tris–Cl, pH 8.0; 25 mM EDTA, pH 8.0; 0.5% (w/v) SDS; 20 µg ml^−1^ RNase A) mixed with the sample by vortexing. Incubation followed at 37 °C for 1 h. Subsequently, 50 µl of Proteinase K (Qiagen, 20 mg ml^−1^ stock) was added. Slow mixing was done by end-over-end rotation 10 times followed by incubation at 50 °C for 3 h interrupted by mixing or by rotating end-over-end 10 times after 1 and 2 h. The resulting viscous lysate was poured into a 50-ml tube. Then, 10 ml of buffer-saturated phenol was added. The lysate was placed on a rotator at 40 rpm for 10 min, during which a very fine emulsion formed, and centrifuged at 3,220*g* for 10 min. The aqueous phase was poured into a new 50-ml tube. Subsequently, 5 ml buffer-saturated phenol and 5 ml chloroform were added. The sample was placed on a rotator at 40 rpm for 10 min during which a very fine milky emulsion formed. The sample was centrifuged at 3,220*g* for 10 min. The aqueous phase was poured into a new 50-ml tube. Then, 4 ml of 750 mM sodium acetate was added. Subsequently, 30 ml of ice-cold ethanol was added followed by 10 gentle end-over-end rotations. Then, the sample was incubated in ice for 1 h. Web-like DNA was collected using a glass hook, while allowing the excess liquid to drop off. DNA was submerged in 70% ethanol and transferred into a 1.5-ml tube, and 1 ml of 70% ethanol was added to the 1.5-ml tube. DNA was centrifuged at 10,000*g* and ethanol was removed. The remaining ethanol was allowed to evaporate at 40 °C for 10 min. DNA was resuspended by adding 150 µl of elution buffer (10 mM Tris–Cl, pH 8.5) and incubating without mixing at 4 °C overnight. DNA was prepared for sequencing using either of the Oxford Nanopore Technologies Rapid Sequencing kits (RAD002 with R9.4.1 flowcell on Mk1B or RAD114 with R10.4.1 on Mk1D). Basecalling was done using the default MinKNOW software.

### Identifying IS-spanning long reads

Synthetic sequences were generated by joining together the sides of the AmpliFinder-predicted amplicon, periamplicon region and the IS element. For the IS element, the entire sequence was used, while for the amplicon and periamplicon, 200 bp of either side was used. The set of six synthetic junctions included the left IS, internal IS, right IS, left no-IS, internal no-IS and right no-IS. Each junction is either an entire IS element (yellow) with two expected 200-bp flanks (grey or cyan), or the same genomic junctions lacking an IS between them. Long reads were aligned using BLAST (version 2.10.1), and a hit was considered spanning the junction if a match was identified covering the entire sequence barring a distance of 50 bp from the ends of the synthetic sequences, allowed to accommodate potential mismatches.

### Identifying amplification-spanning ultra-long reads

Long reads were mapped by BLAST version 2.10.1 to a reference genome. To reduce alignment errors, the reference genome, used as a database to which the query is aligned, was first modified to include SNPs, insertions and deletions, as identified by Breseq analysis of short-read sequencing data of the same isolate. BLAST hits were filtered by limiting the *e*-value < 1 × 10^−4^ and the alignment length longer than 200 bases. Reads were considered amplification spanning if their hits covered at least 1 kb both left and right of the expected amplicon. The hits were then plotted on the reference genome to reveal the repeat pattern, which was visually inspected.

### IS element clustering

The sequences of all IS elements identified in IS-associated amplifications were compared with each other using BLAST (version 2.10.1) with an *e*-value threshold of 1 × 10^−4^. IS elements were clustered by connected component analysis. For enrichment analysis of IS element clusters across amplification types, for each IS cluster and each pairwise amplification-type comparison (flanked versus hemi-flanked, flanked versus unflanked, hemi-flanked versus unflanked), enrichment was assessed with a two-sided Fisher’s exact test using MATLAB’s default fishertest. Significance was determined using a Bonferroni-adjusted *P* value (*P* × number of IS clusters × 3 comparisons).

### ARG identification and enrichment test

Amplicons from experiments in which bacteria were selected for antibiotic resistance (Supplementary Table 1) were aligned to the Antibiotic Resistance Database (retrieved 12 September 2022^99^) using BLAST (version 2.10.1). ARGs were called for BLAST hits covering >0.8 the length of an Antibiotic Resistance Database entry with >0.8 identity. For BLAST hits with an overlap larger than 0.1 of the hit length, only the higher quality hit was considered. For each amplicon, the number of ARG hits was considered. To measure the enrichment of each amplicon to ARGs, the reference genome was sampled 1,000 times for regions of the same length and the number of expected ARGs per amplicon was then calculated as the mean of the random genomic regions. For each type of amplification (flanked, hemi-flanked and unflanked), enrichment was defined as the total number of observed ARGs in all amplifications of this type divided by the total number expected for random amplicon positions. To find, for each amplification type, the significance of enrichment, we bootstrapped the amplicons in each amplification type and assigned a *P* value based on the fraction of resampling resulting in the same or higher ARG enrichment. For pairwise comparison of enrichment between two amplification types, the ratio between mean enrichment (observed divided by expected) of two amplification types is calculated and compared with the ratio in a permuted dataset of over 20,000 permutations. The difference in log of enrichment ratios between the two types is used for a two-tailed test where the *P* value is equal to the fraction of permutations leading to a difference equal to or greater than the one of the non-permuted data.

### Measuring evolvability of mutations

For each isolate from the ampicillin adaptation MEGA-plate experiment (Supplementary Table 1), we identified both SNPs (identified by breseq version 0.32.1) and IS-associated amplifications <250 kb (identified by AmpliFinder and manually validated, equivalent to the list found in Supplementary Table 3). For each SNP or IS-associated amplification in each experimental setting, we found the ratio of maximal to minimal MIC among its carriers. A two-sample Kolmogorov–Smirnov test was used to determine whether this ratio differs between the two groups of genetic changes.

### Reporting summary

Further information on research design is available in the Nature Portfolio Reporting Summary linked to this article.

## Supplementary information

Supplementary Information Supplementary Fig. 1.

Reporting Summary

Supplementary Table 1 List of projects.

Supplementary Table 2 List of isolates.

Supplementary Table 3 List of amplifications.

Supplementary Table 4 List of unresolved amplifications.

Supplementary Table 5 ARG enrichment.

Supplementary Table 6 List of nested amplifications.

## Source data

Source Data Fig. 2 Statistical source data.

Source Data Fig. 3 Statistical source data.

Source Data Fig. 4 Statistical source data.

Source Data Extended Data Fig. 3 Statistical source data.

Source Data Extended Data Fig. 4 Statistical source data.

Source Data Extended Data Fig. 5 Statistical source data.

Source Data Extended Data Fig. 6 Statistical source data.

Source Data Extended Data Fig. 8 Statistical source data.

Source Data Extended Data Fig. 9 Statistical source data.

## Data Availability

All short- and long-read data generated as part of this research project have been deposited in the NCBI SRA database under accession codes PRJNA1178432, PRJNA1141233, PRJNA993583 and PRJNA1472953. Raw data for colony-forming unit counts are available via Figshare at 10.6084/m9.figshare.28669331 (ref. ^100^). Source data are provided with this paper.

## Code availability

The AmpliFinder code is available via GitHub at https://github.com/Technion-Kishony-lab/amplifinder.
