# Supplementary material for: Non-canonical gene amplifications facilitate adaptive evolution in bacteria
Source: Nat Microbiol. 2026 Jul 6;11(8):2201–12. doi: 10.1038/s41564-026-02415-2 (PMC13423834; doi:10.1038/s41564-026-02415-2)
Supplement: Supplementary file 1 — Supplementary Fig. 1. [file 41564_2026_2415_MOESM1_ESM.pdf]

nature microbiology

Supplementary information https://doi.org/10.1038/s41564-026-02415-2

Non-canonical gene amplifications facilitate adaptive evolution in bacteria

In the format provided by the authors and unedited

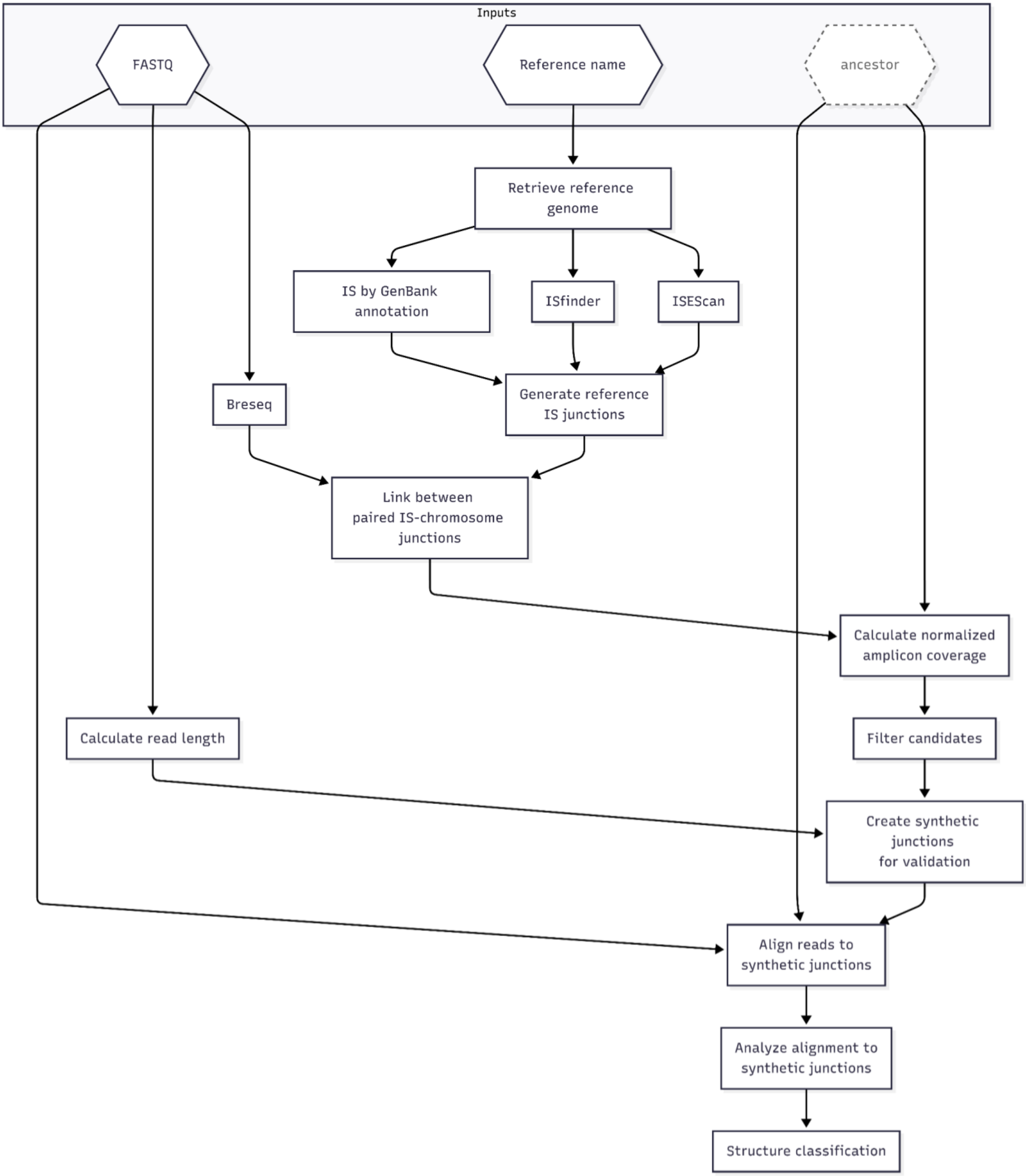

Supplementary Fig. 1: Flowchart illustrating the overall architecture and main processing steps of AmpliFinder. The diagram summarizes the workflow and logic leading from input data (FASTQ files, reference name and an optional ancestor (dashed hexagon)) to final amplification structure classification. For a detailed description of steps, also see documentation of the available AmpliFinder package. Flowchart generated by the Mermaid diagramming and charting tool (https://github.com/mermaid-js/mermaid).
